# Long-Range Traffic Monitoring Based on Pulse-Compression Distributed Acoustic Sensing and Advanced Vehicle Tracking and Classification Algorithm

**DOI:** 10.3390/s23063127

**Published:** 2023-03-15

**Authors:** Iñigo Corera, Enrique Piñeiro, Javier Navallas, Mikel Sagues, Alayn Loayssa

**Affiliations:** Institute of Smart Cities and Department of Electrical, Electronic and Communications Engineering, Universidad Pública de Navarra, 31006 Pamplona, Spain; inigo.corera@unavarra.es (I.C.); enrique.pineiro@unavarra.es (E.P.); javier.navallas@unavarra.es (J.N.); alayn.loayssa@unavarra.es (A.L.)

**Keywords:** traffic monitoring, vehicle classification, distributed acoustic sensing, optical pulse compression, optical time domain reflectometry

## Abstract

We introduce a novel long-range traffic monitoring system for vehicle detection, tracking, and classification based on fiber-optic distributed acoustic sensing (DAS). High resolution and long range are provided by the use of an optimized setup incorporating pulse compression, which, to our knowledge, is the first time that is applied to a traffic-monitoring DAS system. The raw data acquired with this sensor feeds an automatic vehicle detection and tracking algorithm based on a novel transformed domain that can be regarded as an evolution of the Hough Transform operating with non-binary valued signals. The detection of vehicles is performed by calculating the local maxima in the transformed domain for a given time-distance processing block of the detected signal. Then, an automatic tracking algorithm, which relies on a moving window paradigm, identifies the trajectory of the vehicle. Hence, the output of the tracking stage is a set of trajectories, each of which can be regarded as a vehicle passing event from which a vehicle signature can be extracted. This signature is unique for each vehicle, allowing us to implement a machine-learning algorithm for vehicle classification purposes. The system has been experimentally tested by performing measurements using dark fiber in a telecommunication fiber cable running in a buried conduit along 40 km of a road open to traffic. Excellent results were obtained, with a general classification rate of 97.7% for detecting vehicle passing events and 99.6% and 85.7% for specific car and truck passing events, respectively.

## 1. Introduction

Effective management of transport networks and the development of intelligent transportation systems rely heavily on road traffic monitoring. Information about vehicle location, count, speed, classification, and traffic density is essential for this purpose. A number of sensing technologies have been developed over the years to provide such information, which can be categorized into intra-vehicular and urban sensing technologies. The latter relies on sensors that are either installed in the pavement (such as inductive loops, pneumatic tube sensors, and passive magnetic sensors) or positioned above ground on supporting structures (such as radars, video cameras, and microwave detectors) [1,2,3]. Although most of these are mature technologies that have already demonstrated their accuracy and robustness, their ability to provide information is limited to specific locations on the road. Therefore, the monitoring of large areas requires the installation of a considerable number of sensors, resulting in high installation, maintenance, and energy consumption costs. Pavement-based sensors can also cause traffic disruption during installation and repair, while other sensor types pose privacy concerns that require careful management [1].

Distributed acoustic sensors (DAS) are increasingly being considered for traffic monitoring due to their unique capabilities, which stem from their distributed nature and set them apart from other technologies [4,5]. These optical sensors take advantage of optical interferometric effects to detect and quantify small vibrations in the fiber resulting from external excitation. In this way, a regular single-mode optical fiber cable of tens of kilometers becomes a dense seismic recording array of vibration sensors, which can be simultaneously interrogated at very high sampling rates [6,7]. Moreover, within the cables, DAS measurement can be performed by using dark (unused) fibers or by transmitting the sensing signals on different optical wavelength channels to the ones used for communications on lit (operating) fibers [5,8].

DAS technology can be applied for traffic monitoring using either purpose-installed optical fiber cables [9,10,11,12,13] or pre-existing optical fiber cables owned by public administrations or telecommunication operators that are already installed along roads [14,15,16]. Using pre-existing optical cables, which are usually installed inside a duct, can help avoid installation and maintenance costs while speeding up the implementation time of this technology. However, this approach imposes more demanding requirements on the DAS interrogation unit, as the sensitivity of the fiber optic cable to vibrations induced by the vehicles is lower than in the case of deploying optical fibers specifically installed with ad hoc asphalt-embedding methods that place the fiber in close mechanical contact with the road.

The first demonstrations of DAS sensors for traffic monitoring that used existing telecommunication infrastructures were just proof-of-concept experiments in which passing vehicles were detected by the vibration induced on nearby fibers running along the road, but there was no emphasis on the measurement range or accuracy, and they used just manual signal processing. However, recent, more advanced works have shown that automated DAS-based traffic monitoring systems can provide statistical data on vehicle count, traffic density, and even vehicle speed estimation [5,17,18,19]. Huang et al. demonstrated that vehicle count, density, and speed could be detected with good accuracy using a fiber optical cable where high-speed data were simultaneously transmitted [5]. Lindsey et al. performed continuous DAS recordings using an automatic template-matching vehicle detection algorithm based on roadbed strain to analyze traffic pattern changes during the pandemic [17]. Similarly, Wang et al. took advantage of the array nature of DAS traces to estimate the traffic volume and mean speed of the vehicles applying beamforming techniques [18]. A more recent work has shown the feasibility of implementing a real-time processing workflow that enables statistical analysis of real traffic situations in large-scale urban environments [19]. All the previous results have demonstrated the outstanding capabilities of DAS traffic monitoring but are limited to obtaining statistical data on vehicle count, traffic density, and vehicle speed. Nevertheless, Catalano et al. went a step further, demonstrating that good accuracy and automation are possible for vehicle classification using a fiber optic cable installed inside a duct pipe using the air-blowing method [4]. However, the technique relies on an optical interrogation setup based on direct detection, which limits both the vehicle classification accuracy and the measurement range, which is constrained to short distances. Altogether, there is still ample room to enhance these systems in terms of their optical setups and signal processing algorithms in order to improve their sensitivity, range, and accuracy in classifying vehicles.

This paper introduces an automated DAS-based traffic monitoring solution that enables long-range and high-accuracy vehicle count and classification using existing telecommunication infrastructures. The proposed system utilizes the optical pulse compression (OPC) technique in a coherent optical time-domain reflectometry (COTDR) setup, which provides higher sensitivity and range than previous approaches and that, to our knowledge, is the first time that is demonstrated in the context of traffic-monitoring DAS. We also introduce a new vehicle detection and tracking algorithm based on a novel transformed domain that enhances the accuracy in the determination of their spatio-temporal trajectories. Finally, we demonstrate a vehicle classification stage based on support vector machines (SVM) that takes advantage of the linearity of the OPC-COTDR measurements with the excitation induced in the fiber. The SVM relies on the unique “DAS signature” of each vehicle type, including cars, trucks, and motorbikes, and is also used as an aid to identify false positives from the detection stage. Our system has been tested in a real-world environment, using a 40-km telecommunication fiber cable that runs along a road open to traffic.

## 2. Optical Pulse Compression DAS for Traffic Sensing

**Figure 1:**
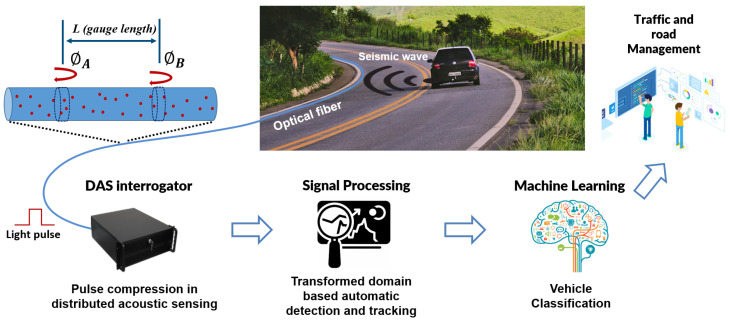
Schematic description of the automatic traffic monitoring system based on DAS.

Figure 1 schematically depicts the configuration of our traffic monitoring system. Vehicles circulating along the road generate seismic waves that are transmitted through the ground and reach an optical fiber telecommunications cable that is typically installed along the road in a conduit or with direct burial. This acoustic wave generates an excitation of the fiber that changes the local optical path length. This excitation is measured using reflectometric methods with a so-called DAS interrogator that injects pulsed laser signals into the fiber and receives and processes the backscattered optical waves. This backscattering is due to the Rayleigh scattering effect in the fiber by which a very small fraction of the light propagating in the fiber is reflected at every position due to fluctuations in refractive index in a scale much smaller than the wavelength of the light.

Different types of DAS interrogator setups can be used for traffic monitoring applications. They differ in the method that is used to measure the vibrations in the fiber [6]. For instance, direct-detection phase-sensitive optical time-domain reflectometry sensors have been deployed for vehicle detection and classification [4,20]. These are based on detecting changes in the amplitude of the backscattered signal from a given position in the fiber as a result of the alterations in the optical path lengths of the interfering signals reflected by the scattering centers that are illuminated by a propagating pulse. However, these sensors present a highly nonlinear response, which compromises the classification of a particular vehicle from the vibration signal obtained as a result of the excitation produced in the fiber by the seismic waves generated by that vehicle.

Other schemes take advantage of coherent optical detection to recover the full optical field (amplitude and phase) of the backscattered signal. In differential-phase COTDR (see the schematic drawing in Figure 1), the phase difference of the optical signals backscattered from two sections of the fiber, A and B, each with a length given by half the pulse duration and separated by the gauge length of the measurement, *L*, is given by [6]:

(1) $$\Delta \varphi =\frac{4\pi n}{\lambda }\xi \Delta L\left(t\right)+\phi_{B}-\phi_{A}=\Delta \varphi \left(t\right)+\varphi$$

where ΔL(t) is the change in the gauge length induced by strain in the fiber, ξ is a correction in the optical path length change that accounts for the strain-optical effect, *n* is the refractive index of the fiber, λ is the wavelength of the optical source, and $\phi_{A}$ and $\phi_{B}$ are the intrinsic random phase shifts of the reflection from A and B. In (1), Δφ(t) and $\varphi \equiv \phi_{B}-\phi_{A}$ are defined to group the excitation and the intrinsic phase terms. Notice that, as long as the intrinsic phase term dependence on the excitation is negligible, the recovered phase difference is proportional to the change in the optical path length inside the gauge length, which, in turn, is linearly related to the vibration induced by the vehicles in that position of the fiber. As a consequence of this linear behavior, differential-phase COTDR setups are better suited for vehicle classification purposes. Moreover, differential-phase COTDR setups have another intrinsic advantage for long-range applications because the measured backscattered signal is proportional to the amplitude of the optical field instead of its power. Therefore, the detected signal is attenuated with the distance at half the rate than in direct-detection methods [4].

We deploy, for the first time in the field of traffic monitoring, to our knowledge, a refinement of the differential-phase COTDR setup in which OPC is implemented [21] in this work. OPC is based on the same pulse-compression principles that have been used for decades in radar systems but translated to the optical domain. Our interest in OPC-COTDR lies in the fact that it is the sensor configuration that, to date, has demonstrated the longest measurement range in purely passive links without additional distributed amplification [22,23]. In OPC-COTDR, long-duration (high energy) and high time-bandwidth product waveforms are launched into the fiber, and the backscattered signals are processed upon reception with matched filters to produce narrow effective pulse widths [21]. Therefore, the compressed pulse amplitude is proportional to the energy of the waveform, providing an enhancement in the measurement signal-to-noise ratio (SNR) that naturally leads to long-range and high-resolution measurements. This SNR improvement is, in principle, proportional to the increased duration of the compression waveform compared to the use of a simple pulse.

In the following, we derive the basic expressions that describe the operation of a DAS sensor based on pulse compression [24,25]. The optical signal that is launched into the fiber in an OPC-COTDR sensor can be expressed as:

(2) $$E_{\mathrm{IN}}\left(t\right)=E\left(t\right)e^{j\phi (t)}e^{j\omega_{0}t}$$

where E(t) is the pulse compression waveform, which can be, for instance, a linear frequency modulated (LFM) pulse or a coded pulse sequence, such as Golay [26] or PPA [27], $\omega_{0}$ is the center radial optical frequency, and ϕ(t) represents the phase noise of the laser source. The total optical signal reflected from the fiber is detected in a coherent receiver with full I/Q demodulation whose local oscillator is a sample of the original laser source. The resultant complex signal is:

(3) $$A\left(t\right)=\int_{0}^{T}r\left(\tau_{rt}\right)E\left(t-\tau_{rt}\right)e^{j[\phi \left(t-\tau_{rt}\right)-\phi \left(t\right)]}e^{j\omega_{0}\tau_{rt}}d\tau_{rt}$$

where $r\left(\tau_{rt}\right)$ is the random Rayleigh backscattering complex reflection coefficient for a position in the fiber with a roundtrip delay $\tau_{rt}$ to the origin, and *T* is the roundtrip delay to the end of the fiber. Notice that the detected signal includes a phase noise difference term due to the difference in propagation delay between the backscattered signals and the local oscillator paths. The received signal, A(t), is then digitally cross-correlated with E(t) to obtain the “compressed response”, i.e., the measured complex backscatter profile of the sensing fiber, $\tilde{r}\left(\tau \right)$:

(4) $$\tilde{r}\left(\tau \right)=\int_{0}^{T}r\left(\tau_{rt}\right)e^{j\omega_{0}\tau_{rt}}\int_{-\infty }^{+\infty }E^{*}\left(t-\tau \right)E\left(t-\tau_{rt}\right)e^{j[\phi \left(t-\tau_{rt}\right)-\phi \left(t\right)]}dtd\tau_{rt}$$

where the time variable has been changed to τ≡2z/v to denote the roundtrip time to a position *z* in the fiber with *v* group velocity.

If we assume that we are in the ideal situation in which the phase noise term can be neglected ($\phi \left(t-\tau_{rt}\right)-\phi \left(t\right)\approx 0$), the compressed response becomes:

(5) $$\tilde{r}\left(\tau \right)=\int_{0}^{T}r\left(\tau_{rt}\right)e^{j\omega_{0}\tau_{rt}}R_{EE}\left(\tau -\tau_{rt}\right)d\tau_{rt}$$

where $e^{j\omega_{0}\tau_{rt}}$ is just a linear propagation delay phase term and $R_{EE}\left(t\right)$ refers to the auto-correlation of the transmitted compression waveform signal, E(t). Therefore, with a well-chosen compression waveform, where $R_{EE}\left(t\right)$ is a narrow function of time, the measured fiber response becomes $\tilde{r}\left(\tau \right)\approx r\left(\tau \right)e^{j\omega_{0}\tau }$, i.e., the complex Rayleigh reflection coefficient, including the propagation delay. Then, measurements according to (1) can be performed using the phase of the compressed response.

**Figure 2:**
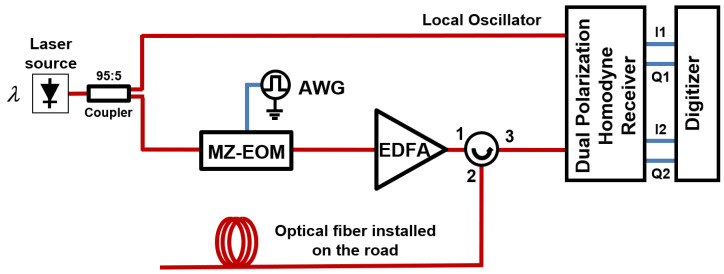
Experimental setup of an OPC-COTDR DAS interrogator.

The experimental setup that we used to implement an OPC-COTDR sensor interrogator in our system is shown in Figure 2. In this setup, the output of a narrow linewidth laser source is split into two branches. One branch is directly connected to the local oscillator input of a homodyne receiver that comprises a 90${}^{\circ }$ dual-polarization optical hybrid and four balanced detectors so that phase and polarization diversity is provided. The other branch is fed to a Mach–Zehnder electrooptic modulator (MZ-EOM) that, in the experiments, is used to translate to the optical domain the LFM pulses that are generated by an arbitrary waveform generator (AWG). The MZ-EOM is biased at minimum transmission so that it generates optical double-sideband suppressed-carrier modulation. Therefore, in the optical domain, two simultaneous LFM pulses (one at each side of the optical carrier) are obtained. In our system, we used LFM pulses with 50-MHz peak-to-peak frequency deviation centered at 50 MHz resulting in a spatial resolution after the pulse compression in the reception of 2 m. The output of the MZ-EOM is amplified in an EDFA and launched into the sensing fiber. Finally, the backscattered signal from the fiber is detected in the homodyne receiver and then digitized and post-processed in a computer.

The first processing applied to the raw measurement data is pulse compression, in which the received signal is digitally cross-correlated with the ideal LFM waveform as it is described above. The next stage deals with the mitigation of signal fading, which is intrinsic to the Rayleigh distribution of the modulus of $r\left(\tau \right)$. This is required to ensure that the calculations using (1) are not distorted by noise contributions. The signal fading method employed involves the application of the rotated-vector-sum method [28] combined with a spatial moving average. Further, the signals obtained from each sideband, which have independent fading statistics, are coherently combined. Finally, the phase difference between consecutive positions separated by the gauge length is computed.

**Figure 3:**
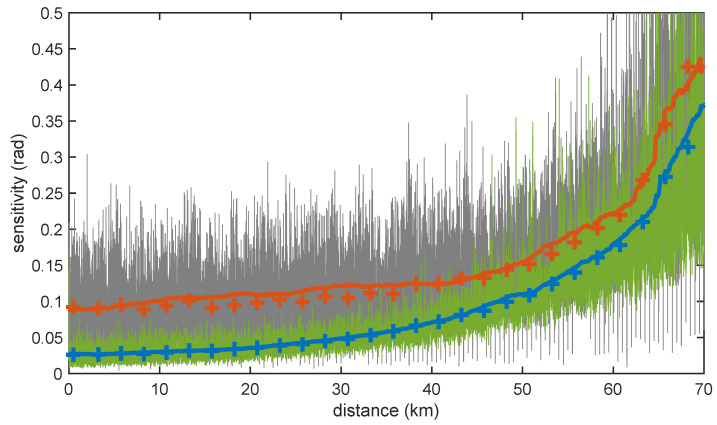
Sensor sensitivity along a 70-km measurement range using LFM OPC with 2-m spatial resolution and 6-m gauge length for two different laser sources: a 100-Hz linewidth NKT (green line) and a 4.1-kHz linewidth RIO modules (grey line). The figure also shows the measured (lines) and numerically calculated from the theoretical model (symbols) smoothed sensitivity data (blue and red for the NKT and the RIO modules, respectively).

To fully design our interrogator system to provide the performance required by traffic monitoring, it was necessary to define its operational parameters attending to the different impairments and noise sources that degrade the measurements. A very important impairment to consider in OPC-COTDR setups is the effects of phase noise of the laser source. In the derivation of (4), we neglected the differential phase noise term, but strictly, this can be just performed for short fiber links using very narrow-linewidth lasers. For longer ranges, the presence of this phase-noise-induced term can severely degrade the performance of OPC-COTDR sensors, as we have demonstrated in a recent study in which we have also derived a theoretical model for this effect [24]. The degradation brought by the laser phase noise increases with the linewidth of the optical source deployed. This is illustrated in Figure 3, where we compare the experimentally measured sensitivity of our sensing setup deploying two different 1550-nm lasers: an NKT Kosheras E15 with 100-Hz nominal linewidth and an RIO Orion module with 4.1-kHz linewidth for a 70-km sensing link when 4-μs LFM pulses were launched into the fiber. The sensitivity of the sensor for each position in the fiber was calculated as the standard deviation of 64 realizations of the differential phase measurement for that location while the fiber under test was placed inside an acoustic- and vibration-isolating enclosure. Notice that the sensitivity presents rapid changes in position due to the residual variation of the received signal amplitude from each location after fading compensation. Therefore, the figure also depicts smoothed data for each laser after the application of a moving average to better highlight the basic trends. For short distances, where the detection noise is relatively low, and hence the SNR of the detected signal is still high, the sensitivity of the sensor is mainly determined by the deleterious effects of phase noise on the pulse compression, resulting in worse sensitivity for the RIO module than for the NKT due to the larger linewidth of the former. However, as we progress along the fiber and the backscattered signals are increasingly attenuated, additive noise in detection becomes the primary factor that undermines the performance of the sensor and hence, the sensitivity degradation tends to equalize for both laser types. Figure 3 also depicts the results of numerical simulations using an enhanced version of our theoretical model for OPC-COTDR sensors that contemplate the effects of the phase noise of the laser as well as additive white Gaussian noise [24]. Note the excellent agreement between experimental and theoretical model results that suggest that the latter can be confidently used in the design stage of OPC-COTDR sensors.

**Figure 4:**
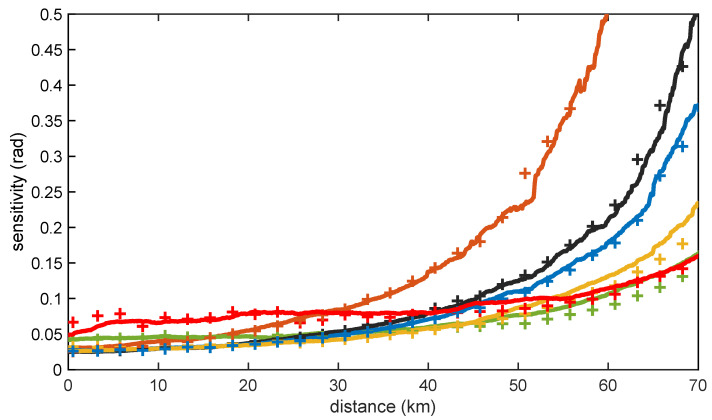
Sensitivity along a 70-km measurement range using LFM OPC with 2-m spatial resolution and 6-m gauge length for the 100-Hz linewidth NKT laser. Numerical (symbols), as well as experimental results (solid lines), are shown for different LFM pulse durations: 1-μs (dark orange); 3-μs (black); 4-μs (blue); 7-μs (orange); 15-μs (green); and 30-μs (red). A moving average was applied to smooth the data.

Apart from the linewidth of the laser source, the other parameter that we can control to optimize performance is the duration of the compression waveform that is launched in the fiber. There is a trade-off between SNR enhancement and phase-noise-induced degradation in the determination of this duration. The longer the compression waveform, the more energy it has and the larger the SNR enhancement brought by the application of OPC. However, as we have recently demonstrated, the sensitivity of an OPC-COTDR sensor degrades with the duration of the compression waveform due to the effects of the phase noise of the laser [24]. This is graphically highlighted in Figure 4, where the experimentally measured and theoretically calculated (smoothed) sensitivities for our LFM-OPC interrogator are depicted for different durations of the LFM compression waveform used. The two expected trends can be noticed in these results. Longer LFM pulses lead to better sensitivity for long-distance measurements for which the backscattered signal becomes faint, and the additive detection noise becomes the prevailing impairment. However, for shorter lengths, where phase-noise effects are predominant, the sensitivity degrades with the LFM duration.

Therefore, the pulse duration for a given fiber link length can be found by increasing the pulse duration till the desired sensitivity is obtained at the end of the link. For this duration, the deleterious effects of phase noise for short distances are reduced while maximizing the benefits gained from pulse compression at the far end of the fiber. The most relevant DAS parameters that were used to perform the traffic monitoring measurements presented in this paper are summarized in Table 1. Moreover, the sensitivity of the optical interrogator is described by the green line in Figure 3, as 4 μs LFM compression waveforms were used for the measurements.

## 3. Automatic Traffic Detection and Tracking

**Figure 5:**
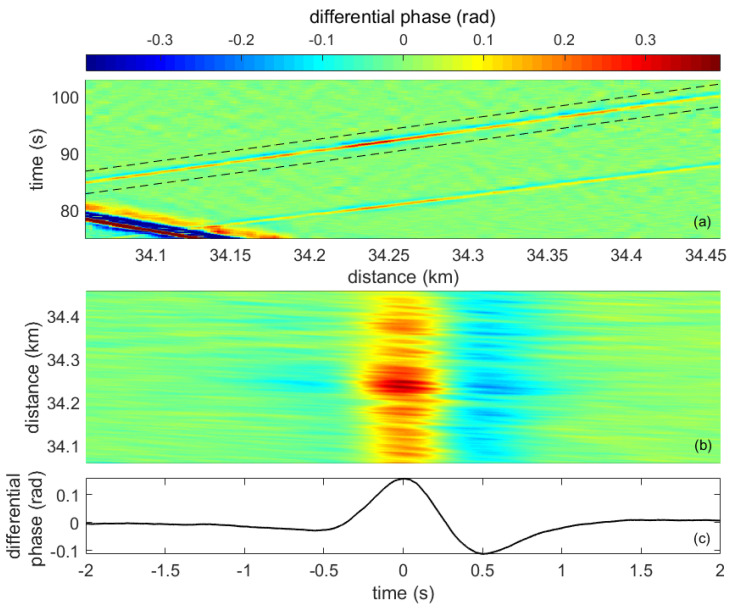
Vehicle passing event represented by: (**a**) the heat-map representing the differential phase measurements for a given time-distance window, where a vehicle signature signal trajectory can be identified, (**b**) a two-dimensional crop of the differential phase signal of ±2 s around the trajectory; and (**c**) the vehicle signature trace, an averaged version of the former signal representing the vehicle signature in the time-axis.

**Figure 6:**
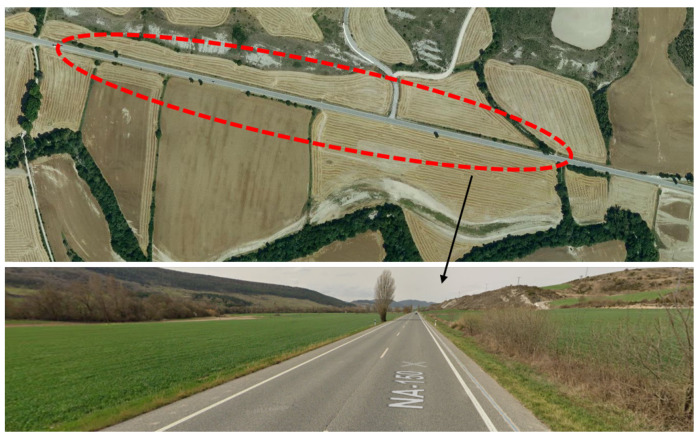
Section of the road used for traffic measurements. ©2023 Google, CNES/Airbus, Gobierno de Navarra-Instituto Geográfico Nacional de España, Maxar Technologies.

Once optimized for the application, our sensor configuration produced high-sensitivity and high-resolution vibration measurement signals suitable to be used as input for the vehicle detection and tracking algorithm. Figure 5 depicts an example of such signals obtained from a spare dark fiber on a 40-km telecommunication cable, which is currently used by the Gobierno de Navarra to connect two of its premises. Specifically, the measurements come from the 1 km section of the road displayed in Figure 6, which is at a distance of 34 km from our interrogator. Nevertheless, notice that all the measurements shown in this paper were performed in a distributed manner over the entire length of the fiber cable; however, only specific sections are highlighted in the figures for the sake of clarity. The fiber-optic cable is installed buried in the shoulder of a two-way road inside a micro duct using the conventional air-blowing method at a depth of 40 cm below the road surface. The distance of the cable from the road center line is approximately 5 m. Vehicles circulating on the road generate small vibrations on the pavement, which propagate through the ground, reaching the optical fibers inside the cable. These vibrations are detected by the DAS sensor, resulting in measurements such as the one shown in Figure 5a. This is a so-called heat-map graph type that displays the measurement of the differential phase, which is proportional to the detected vibration in the fiber, along a specific section of the road over a given time. The spatiotemporal trajectory of a particular vehicle is highlighted in the figure by two black dashed lines. Figure 5b displays a zoom of the signal in a time axis relative to the vehicle trajectory. From this signal, the vehicle’s signature trace shown in Figure 5c can be calculated by taking the median of the measurement in the distance dimension. This signature can be regarded as an averaged signal representing the impact of a vehicle when passing across an arbitrary position on the road. Notice that these measurements, and the rest of the measurements in the paper, were obtained using a spatial moving average of 6 m to mitigate fading and a gauge length of 6 m. As shown in (1), the differential phase is linearly related to the vibration induced by the vehicles in a given position of the fiber. It is well known that optical DAS interrogators analyzing the vibration of optical fibers buried along the roads can detect the two fundamental components of these vibration signals [17]. On the one hand, we have the high frequency (>3 Hz, approximately) anthropogenic surface waves radiated by vehicles. On the other hand, a low-frequency quasi-static or geodetic signal is induced by the road’s response to point-load vehicle movements. This component provides information about the loading and unloading of the road near the fiber and will be used to identify the individual vehicle’s DAS signature. A preliminary analysis showed that the spectral components of the measured dynamic signals were below 50 Hz. Hence, the pulse repetition frequency was set to 200 Hz, more than enough to ensure that no aliasing effects were present. Then, the differential phase data were bandpass filtered from 0.3 to 3.5 Hz, where the fundamental quasi-static components were found to lie.

Measurements such as those in Figure 5 are used in the final stage of our system when we proceed with the classification of vehicles. However, to have a completely automated traffic monitoring scheme, we first need to identify the vehicles and provide their spatial position along the road as a function of time. To achieve this goal, we devised a traffic detection and tracking algorithm that comprises two steps: local segment detection and segment tracking.

**Figure 7:**
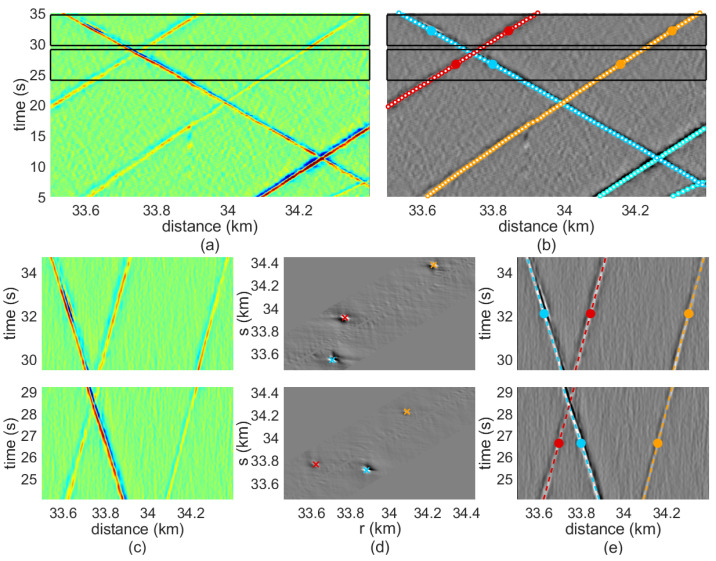
(**a**) Measured vibration intensity and (**b**) results of the vehicle detection and tracking algorithm. (**c**) Detail of the measured vibration intensity (**d**) transformed-domain and (**e**) resulting segments after segmentation for the two time-distance blocks marked with black square boxes in (**a**).

The segment detection method is illustrated with the example measurement in Figure 7, which highlights the differential phase detected by the DAS sensor corresponding to the 1-km section of the road during a 30 s interval. When considering short time intervals, we can assume that vehicles are moving at a constant velocity, implying a locally linear trajectory in the DAS recording. Drawing from this concept, the method operates by partitioning the recorded signal into discrete processing blocks that correspond to brief temporal intervals. This approach enables the identification of linear segments that correspond to the motion of vehicles within each processing block. In this example, the processing blocks comprise a period, Δt, of 5 s. Blocks are calculated every δt= 0.35 s so that there is a temporal overlapping between consecutive blocks of 4.65 s. As an example, two non-consecutive blocks are marked with black square boxes in Figure 7a and are shown in more detail in Figure 7c.

The segment detection algorithm applied at each block relies on a newly proposed transformed domain, in which each point corresponds to a specific straight segment in the normal non-transformed recording domain. Detecting vehicle segments corresponds to detecting local maxima in this new domain.

This Transform is an evolution of that proposed in [12] and can be considered as an extension of the Hough Transform that operates with non-binary valued signals. Let $F\left(t_{n},d_{k}\right)=F\left[n,k\right]$ be the differential phase of a certain NxK processing block in which segment detection is to be applied, where $t_{n}$ represents the time instants within the block time interval, and $d_{k}$ is the spatial position along the optical fiber, with 1≤n≤N and 1≤k≤K being the corresponding temporal and spatial samples. The first step is to compute the Transform $M\left(r_{i},s_{j}\right)=M\left[i,j\right]$ of the processing block at each point of the transformed domain. This computation essentially consists of calculating the median of the set of sample values of the block $F\left(t_{n},d_{k}\right)$ within a certain straight segment defined by the parameters of the transformed domain $\left(r_{i},s_{j}\right)$, where $r_{i}$ is the starting spatial position of the segment at the block’s starting time, and $s_{j}$ is the ending position of the segment at the block’s ending time. Hence, the segment in the signal domain comprises a set of time-space tuples $\left(t_{n},d_{n}\right)$, where times are given by the sampling instants $t_{n}$, and spatial positions are computed as:

(6) $$d_{n}=r_{i}+\frac{s_{j}-r_{i}}{\Delta t}t_{n}\;$$

To adapt it to a discretized spatial domain, a rounding operation can be applied over the segment positions $d_{n}$ so that each of them exactly corresponds to a certain sampling position of the signal $d_{k}$. Thus, $d_{n}\leftarrow d_{k}$, such that it minimizes $\left|d_{n}-d_{k}\right|$. The median signal values within the segment, i.e., the Transform value at $\left(r_{i},s_{j}\right)$, are given by

(7) $$M\left(r_{i},s_{j}\right)=\mathrm{median}\left[{\left\{F\left(t_{n},d_{n}\right)\right\}}_{n=1}^{N}\right]$$

The sampling resolution of the transformed axes, $r_{i}$ and $s_{j}$, is set to 1.8 m. An additional restriction is imposed on the points of the transformed domain to be computed: only those points for which the segment represents a moving velocity in the range [2, 60] m/s are computed.

Once the transformed map $M\left(r_{i},s_{j}\right)=M\left[i,j\right]$ is obtained, the local maxima of the map are extracted. Note that the locations of these maxima correspond to segments of the signal in which the median amplitude value is higher, implying a high probability that these segments are related to the passage of a moving vehicle. Therefore, after computing a segment detection stage for a certain processing block, a set of local maxima in the transformed domain ${\left\{\left(r_{l},s_{l}\right)\right\}}_{l=1}^{L}$ is available (marked with colored crosses in Figure 7d), where *l* indexes the different detected and validated locations. These specific straight segments in the non-transformed domain are shown in Figure 7e, where the colors of each trajectory correspond to those of the local maxima in Figure 7d.

Once local segments are detected in each processing block, the next step is to apply segment tracking to join these segments among the different blocks so that arbitrarily large trajectories corresponding to detected moving vehicles are obtained. The input of the tracking algorithm is the set of local maximum points in the transformed domain ${\left\{p_{l}^{m}\right\}}_{l=1}^{L_{m}}={\left\{\left(r_{l}^{m},s_{l}^{m}\right)\right\}}_{l=1}^{L_{m}}$ detected at each processing block, where *m* indexes the different processing blocks, with $L_{m}$ as the number of points of the *m*th block. The proposed tracking algorithm dynamically initiates, expands, and ends trajectories as the sets of points of new blocks are being considered. Trajectories are defined as sequences of points in the transformed domain, where each point belongs to a different processing block. In this way, the *q*th trajectory is denoted as ${\left\{w_{q}^{m}\right\}}_{m={m_{\mathrm{ini}}}_{q}}^{{m_{\mathrm{end}}}_{q}}={\left\{\left({r_{w}}_{q}^{m},{s_{w}}_{q}^{m}\right)\right\}}_{m={m_{\mathrm{ini}}}_{q}}^{{m_{\mathrm{end}}}_{q}}$, where ${m_{\mathrm{ini}}}_{q}$ and ${m_{\mathrm{end}}}_{q}$ are the indexes of the processing blocks in which the trajectory begins and ends; and $\left({r_{w}}_{q}^{m},{s_{w}}_{q}^{m}\right)$ is the point location in the transformed domain for the trajectory *q* in the block *m*.

Each point of the first processing block gives rise to the generation of a new trajectory, which is initialized containing only the point under consideration. In an iterative procedure, the points of new processing blocks are joined to the already existing trajectories using a distance criterion. This process also enables the generation of new trajectories, as well as the termination of existing ones, which is a key feature in a real-field environment. Next, for each processing block, the distance between the point of each provisional trajectory *q* in the *m*th processing block (the current endpoint of the trajectory) and each point *l* detected in the m+1th processing block is calculated

(8) $$\mathrm{dist}\left[w_{q}^{m},p_{l}^{m+1}\right]=\sqrt{{D_{r}}^{2}+{D_{s}}^{2}}$$

where

(9) $$D_{r}=\left({r_{w}}_{q}^{m}-r_{l}^{m+1}\right)+\frac{\delta t}{2\Delta t}\left({s_{w}}_{q}^{m}-{r_{w}}_{q}^{m}+s_{l}^{m+1}-r_{l}^{m+1}\right)$$

(10) $$D_{s}=\left({s_{w}}_{q}^{m}-s_{l}^{m+1}\right)+\frac{\delta t}{2\Delta t}\left({s_{w}}_{q}^{m}-{r_{w}}_{q}^{m}+s_{l}^{m+1}-r_{l}^{m+1}\right)$$

The points of block m+1 are joined to the provisional trajectories in an iterative process, in which, at each iteration, the combination $w_{q}^{m},p_{l}^{m+1}$ with the lowest distance gives rise to a new union. This process ends when there is no joint whose distance is less than the maximum allowed distance. Once a trajectory is closed, if its length is shorter than a certain number of points, the trajectory is discarded. Otherwise, it is incorporated into the set of definitive trajectories. Finally, a last step is applied to describe these trajectories as a sequence of time-distance tuples in the non-transformed domain. In this domain, each trajectory represents a sequence of segments among the different processing blocks. Therefore, the time and position in the middle of the segment are chosen as the time-distance coordinate of the trajectory within the segment. Figure 7b represents these automatically extracted trajectories as colored points superimposed onto the grey-scale map of the signal in Figure 7a, showing excellent results.

## 4. Vehicle Classification

The output of the tracking algorithm is a set of trajectories, each of which can be regarded as a vehicle passing event. The next stage in the processing system aims to classify the vehicles according to their size (cars and trucks) and travel direction (westbound and eastbound). Again, the one-kilometer section located around the 34th kilometer of the two-way road shown in Figure 6 was selected to demonstrate the classification capabilities of our solution. A video camera was set up at that location to monitor the whole road section and provide independent verification for our vehicle detection, tracking, and classification solution.

Four different classes were defined for the classification of vehicles: cars westbound (CW), cars eastbound (CE), trucks westbound (TW), and trucks eastbound (TE). To enhance the classification ability of the system, three additional classes have been defined that are specifically introduced to reduce the number of misclassified car and truck trajectories: motorbikes traveling in each direction (MW and ME) and a not-a-vehicle class (NV), comprising all the spurious trajectories coming from the tracking algorithm but not related to any real vehicle passing event. As a consequence, missed motorbike events are not a concern in this context. Hence, the classification system was designed to be able to identify seven different vehicle passing event classes. Before proceeding with the feature extraction from the signals, a vehicle signature trace was automatically extracted from each trajectory following the procedure described in Figure 5.

**Figure 8:**
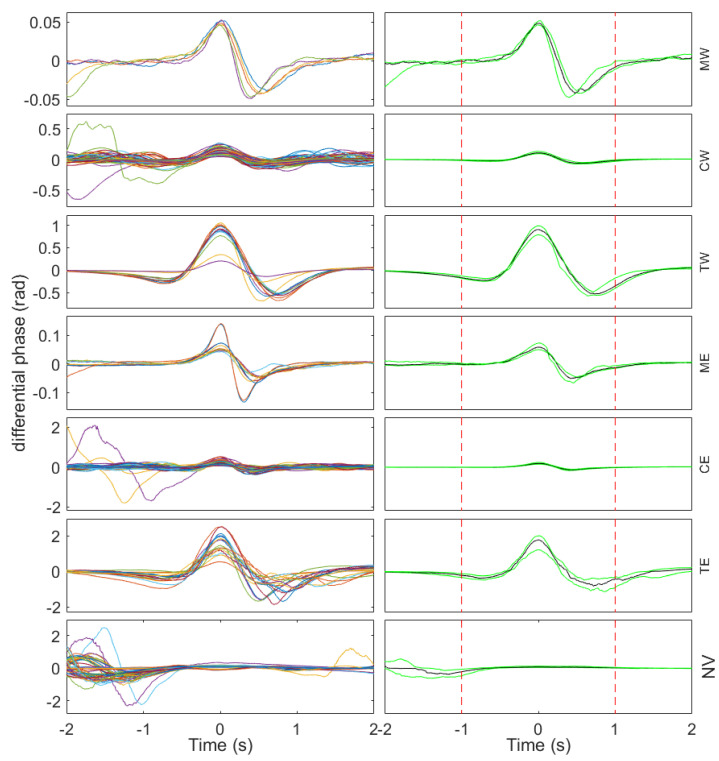
Vehicle signature traces of all the analyzed events grouped by class (left column) and the corresponding median trace (black line) and 25th and 75th percentile signals (green lines) per class (right column). Each row corresponds to one of the analyzed classes: MW, CW, TW, ME, CE, TE, and NV, respectively. The vertical dashed red lines in the left column represent, at each plot, the time instants at −1 s and 1 s.

In the left row of Figure 8, the vehicle signature traces aggregated by their corresponding class is depicted. In the right row of Figure 8, the median from all the signature traces for each class (black line), along with the 25th and 75th percentile limits, are depicted (green lines). It is important to observe that, in general terms, the amplitude of the signature traces corresponding to the motorcycle class is lower than that corresponding to the car class, and in turn, the amplitude for the car class is notably lower than that corresponding to the truck class. Furthermore, the amplitude is generally larger for the traces corresponding to the westbound direction than for those corresponding to the eastbound direction. This is because the fiber is placed on the shoulder next to the westbound direction lane, resulting in a higher sensitivity for the vehicles moving in that direction. Regarding the class of non-vehicles, it is observed that a great amount of signal activity tends to appear on the sides of the interval analysis, especially on the negative time side, something that is not observed for the rest of the classes. It is also observed that only in the non-vehicle class are there signature traces whose value in the center of the interval analysis is negative.

In agreement with the previously described characteristics of the signature traces, feature extraction was designed to extract relevant information from the differences among the vehicle signature traces of the different classes observed in Figure 8. Four features are extracted from the vehicle signature traces:

- Peak-to-peak amplitude: calculated as the decimal logarithm of the peak-to-peak amplitude of the signature trace restricting the analysis to 1 s of the signal around its center (in Figure 8, within the interval delimited by dashed red lines).
- Center amplitude: calculated as the amplitude of the signature trace in its central sample.
- Lateral area: calculated as the decimal logarithm of the summated absolute amplitude values of the signature trace after removing the 1 s central part of the signal.
- Velocity: calculated directly from the trajectory, as the total signed travel of the trajectory on the fast axis, divided by its time span on the slow axis.

**Figure 9:**
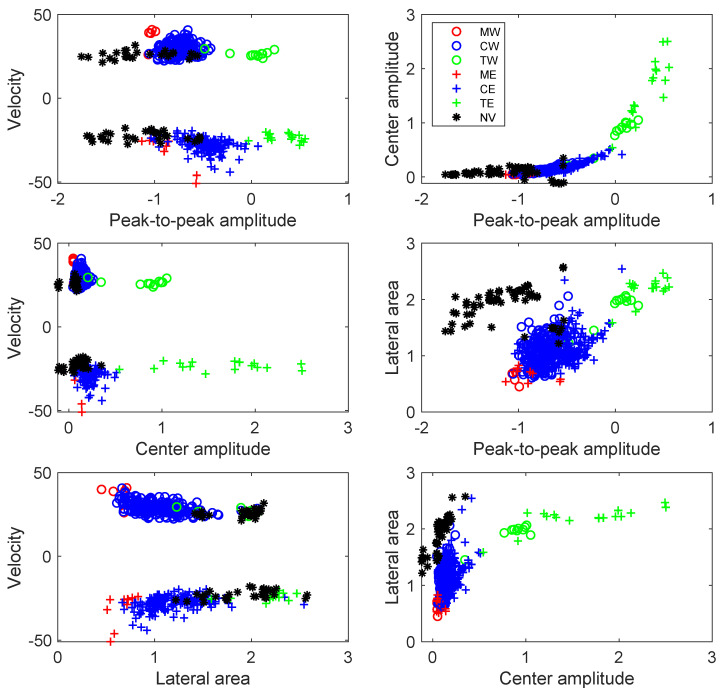
Two-dimensional scatter plot projections of the four-dimensional feature space showing the feature vectors of the seven classes: MW (red circles), CW (blue circles), TW (green circles), ME (red crosses), CE (blue crosses), TE (green crosses), and NV (black asterisks). The plots correspond to the feature combinations: peak-to-peak amplitude vs. velocity; center amplitude vs. velocity; lateral area vs. velocity; peak-to-peak amplitude vs. center amplitude; peak-to-peak amplitude vs. lateral area; and center amplitude vs. lateral area.

Figure 9 presents the two-dimensional scatter plot projections of the four-dimensional feature space. Peak-to-peak amplitude is closely related to the vehicle weight: the greater the weight, the greater the amplitude. For this reason, this feature is useful to separate the car classes (CW and CE), truck classes (TW and TE), and motorcycle classes (MW and ME). The center amplitude is also related to the vehicle weight, providing a similar behavior in terms of separability between classes. However, it is convenient to add this feature to the classification stage, as it provides additional information when separating the non-vehicle class from the rest of the classes. Note that this class is the only one for which several signature traces present a negative value in the center of the interval. Therefore, it provides a straightforward way to separate those negative amplitude cases in the non-vehicle class. Regarding the lateral area, which measures the signal activity on the sides of the window, it provides separability in the non-vehicle class. It must be taken into account that many of the spurious trajectories detected by the segmentation algorithm (labeled as non-vehicles) originated as a consequence of the passage of a truck, which systematically produces a positive after-wave that is detected as a spurious trajectory in the segmentation. This is the reason why the non-vehicle class systematically presents a lot of signal activity on one side of the window. Regarding the velocity, its sign allows a clear separation between classes corresponding to the westbound direction (negative velocity) and those corresponding to the eastbound direction (positive velocity). A set of 512 labeled feature vectors was employed to train and evaluate an SVM classifier. An evaluation was performed under a four-fold cross-validation scheme, with 75% of the observations dedicated to training and 25% of the observations dedicated to testing.

**Figure 10:**
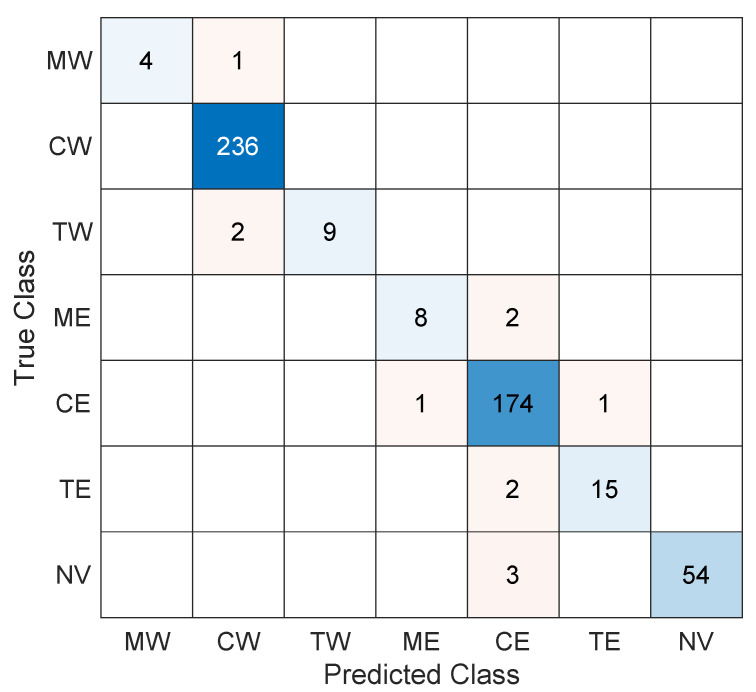
Confusion matrix showing the classification outcome for the different classes. The rows correspond to the true class, and the columns to the predicted class.

The classification results are shown in the confusion matrix of Figure 10. The results show a high classification accuracy of 97.7% for vehicle passing events. When analyzing the confusion matrix, it is important to note that 54 out of 57 not-a-vehicle events are correctly classified. These are spurious trajectory detections left by the tracking algorithm that our classifier can detect and tag to be removed from further analysis. As a consequence, the classification stage can further refine the results from the trajectory tracking algorithm, implementing a second check on its results. In the same vein, incorporating the motorbike classes (in both directions) helps reduce the number of misclassified car and truck trajectories, as the classifier can identify these events as vehicles but not in the car or truck classes. Of the 28 truck events analyzed, keeping in mind the two road directions, 24 have been correctly classified, and 4 have been wrongly classified as cars. However, the erroneous cases correspond to very light trucks, for which the amplitude of the signature trace is small compared to that corresponding to an average truck signature. Regarding the car events, of the total of 453 cases analyzed, 451 have been correctly classified (99.6%), and only 2 were misclassified. Another aspect that could be highlighted is that in no case has a vehicle in one direction of the road been classified as a vehicle in the other direction.

## 5. Conclusions and Future Work

We have demonstrated a long-range and high-resolution automatic traffic monitoring scheme that takes advantage of the enhanced sensing capabilities provided by optical pulse compression in distributed phase-sensitive optical time-domain reflectometry. In addition, a vehicle detection and tracking algorithm has been developed based on a novel transformed domain, enabling high-accuracy non-machine-learning-based traffic monitoring. Moreover, we have also explored the capabilities of the system to provide vehicle classification by implementing a machine-learning stage based on SVM. Moreover, it was found that the addition of a not-a-vehicle class in the classification stage helped identify spurious trajectory detections left by the tracking algorithm. Therefore, this feedback from the classification stage can be used to enhance the results of the trajectory-tracking algorithm.

Regarding the vehicle detection and tracking algorithm, the approach taken in our work has the intrinsic advantage of not requiring a training stage with a massive amount of data, as would be needed in other processing schemes, specifically those relying on deep-learning algorithms. However, it has the limitation of requiring a certain knowledge of the shape of the vibration waveforms induced by the vehicles and captured by the DAS interrogator, as is also the case for the SVM-based classification stage. As a result, our solution may have limitations in scenarios where multiple vehicles generate vibration signals simultaneously, where other non-vehicle-related vibration signals are present or where the coupling of the vibration signals to the fiber is very heterogeneous along the road. This could be the case, for example, in heavy-traffic flow urban scenarios, where applying our algorithmic approach to different road sections may imply a calibration of the recorded DAS signals, which could be performed without a ground-truth reference or even an independent training stage. Moreover, we believe that, in those scenarios, deep-learning approaches could have an advantage over our technique. However, this possibility remains a topic for future research, as it has not yet been demonstrated to the best of our knowledge.

Ongoing work is focused on developing an evolution of the three-stage solution proposed in this paper, which combines OPC-DAS, signal processing, and machine learning technologies for the characterization of the structural integrity of traffic infrastructures (i.e., road roughness level, early detection of cracks, etc.).

## Figures and Tables

**Table 1:** DAS Recording Parameters.

Parameter	Value
Pulse repetition frequency	200 Hz
Gauge length	6 m
Sample spacing	10 cm
ADC sampling frequency	1 Gbps

## Data Availability

Not applicable.

## Abbreviations

The following abbreviations are used in this manuscript:

AWG	arbitrary waveform generator
COTDR	coherent optical time-domain reflectometry
DAS	distributed Acoustic Sensing
LFM	linear frequency modulated
MZ-EOM	Mach–Zehnder electrooptic modulator
OPC	optical pulse compression
SNR	signal-to-noise ratio
SVM	support-vector machines
